# Louse-borne relapsing fever profile at Felegehiwot referral hospital, Bahir Dar city, Ethiopia: a retrospective study

**DOI:** 10.1186/1756-0500-7-250

**Published:** 2014-04-17

**Authors:** Mulat Yimer, Wondemagegn Mulu, Workneh Ayalew, Bayeh Abera

**Affiliations:** 1 Department of Medical Microbiology, Parasitology and Immunology, College of Medicine and Health Sciences, Bahir Dar University, Bahir Dar, Ethiopia; 2 Felegehiwot referral hospital, Amhara Regional Health Bureau, Bahir Dar, Ethiopia

**Keywords:** Louse- borne relapsing fever, Bahir Dar city, Ethiopia

## Abstract

**Background:**

Louse- borne relapsing fever is an acute febrile illness caused by *Borrelia recurrentis* and is transmitted by body lice, *Pediculus humanus corporis*. The disease has occurred as epidemic in different parts of the country.Therefore, the aim of this retrospective study was conducted to assess the LBRF profile for the last four years.

**Methods:**

A retrospective study was conducted on patients with LBRF admitted from 2009–2012 at Felegehiwot referral hospital. The diagnosis was based on both clinical and laboratory methods. Patients with strong clinical suspicion of LBRF and positive for *Borrelia* species in their blood was diagnosed as LBRF cases. Data was collected from all patients with LBRF- like symptoms in their registration book. Data was checked for completeness, coded and analysed using SPSS version 16. P < 0.05 was considered significant for comparison.

**Results:**

Of the 4559 patients admitted with LBRF- like symptoms, 4178 (91.6%) were males and 381 (8.4%) were females. Most of the patients (74.2%) were within age groups 11–20 years. The majority of patients (94.4%) were from urban residence. The overall prevalence of LBRF was 225 (4.9%) and the highest prevalence 171 (5.1%) was observed in age groups of 11–20 years. The association between seasonal variation and prevalence of LBRF showed that more patients with positive for *Borrelia* species were recorded in dry 27 (9.7%) than wet 198 (4.6%) seasons (P < 0.001). Finally, a trend in prevalence of LBRF for the last four years showed that the highest numbers of cases were documented in 2010.

**Conclusion:**

The overall prevalence of LBRF was high and the highest prevalence was observed in young age groups. Moreover, most of the patients with LBRF were from urban dwellers. Therefore, health education should be delivered towards LBRF prevention in the city.

## Background

Relapsing fever (RF) is a vector borne disease caused by *Borrelia* species (body lice in case of louse- borne relapsing fever (LBRF)) and soft ticks in case of tick- borne relapsing fever (TBRF). This acute febrile illness presents with recurrence of characteristic febrile periods lasting for days alternating with afebrile periods [1]. The main manifestation is a recurring fever which coincides with massive numbers of bacteria in the blood and severity ranges from asymptomatic to fatal [2].

LBRF affects millions of people worldwide during the first half of the 20th century, particularly during the world wars [3]. In the past, LBRF had also occurred in large outbreaks in Eritrea, Sudan and Somalia [4]. However, there is no data that depicts the designed controlling mechanism. Transmission of *B. recurrentis* back to humans is accomplished when the louse is crushed while scratching and enters through the abraded skin [5,6] and also facilitated through lice faeces [7].

TBRF in Africa is caused primarily by *Borrelia duttonii*, transmitted by *Orinthodoros moubata* ticks in East and Central Africa, and by *Borrelia crocidurae*, transmitted by *Orinthodoros sonrai* in West Africa. African TBRF is associated with proximity to tick-infested burrows and huts [8,9]. In contrast, LBRF is caused by *B. recurrentis,* which is transmitted by body louse (*P. humanus corporis*). This vector lives in clothes and multiplies when conditions like cold weather, lack of hygiene, or war are present. Its prevalence reflects the socioeconomic level of the society, as it is increasingly described in the poorest populations [10].

LBRF is now an important disease in the highlands of Ethiopia where an estimated 10,000 cases occur annually and affects mostly homeless people living in crowded and unhygienic conditions especially during rainy seasons [11]. It is within the top ten causes of hospital admissions, associated with significant morbidity and mortality [2,8]. For instance, in southern Ethiopia (Hosanna hospital), LBRF admissions comprised 27% of total admissions [2]. Moreover, in south west Ethiopia, 6% of mortality rate was documented [12]. Furthermore, according to the Ethiopian health department report, it is the seventh most common cause of hospital admission and fifth most common cause of death [13] and the disease has occurred as epidemic in different parts of the country [2].

In 2010, it also occurred as epidemic in Bahir Dar city and 2–3 patients on average were admitted at felegehiwot referral hospital per day [data from the registration book]. In spite of having such numbers of admissions, yet there is no information regarding the LBRF profile at this hospital. Therefore, the aim of this retrospective study was to assess LBRF profile for the last four years.

## Methods

### Study period and area

Institutional based retrospective study was conducted on patients with LBRF admitted from 2009–2012 at Felegehiwot referral hospital, Bahir Dar city -Ethiopia. Bahir Dar is situated on the southern shore of Lake Tana, the source of the Blue Nile (or Abay), in what was previously the Gojjam province and now the Amhara National Regional State. The city is located approximately 578 km north-west of Addis Ababa, having an elevation of 1840 meters above sea level. Based on the 2007 Census conducted by the Central Statistical Agency of Ethiopia, it has a total population of 221,991, an increase of 130.90% over the population recorded in the 1994 census, of whom 108,456 are men and 113,535 women [14].

Study participants were all patients admitted with LBRF- like symptoms and the diagnosis was based on both clinical and laboratory methods. According to the standards operational procedure, thick blood film was prepared for each patient and stained with 3% Giemsa for 30 minutes. The slides were examined under 100 X objective using oil immersion objective. Patients with strong clinical presentation of LBRF and positive for *Borrelia* species from their blood was diagnosed as LBRF cases. Finally, the prevalence was determined by dividing all positive cases from all patients admitted with LBRF- like symptoms during the study periods.

### Data analysis

Data was checked for completeness, coded and analysed using SPSS version 16. For descriptive statistics, frequency, percentage and mean were used. While for categorical analysis, Chi-square was used to describe the association between categorical variables and p < 0.05 was considered significant for comparison.

### Ethical consideration

Ethical clearance was obtained from Bahir Dar University, College of Medicine and Health Sciences. Permission letters were obtained from Amhara National Regional State Health Bureau and hospital director office before we commenced for data collection from hospital records.

## Results

Data from hospital patients admitted with LBRF- like symptoms for the last four years (2009–2012) was 4559. Of these, 4178 (91.6%) were males and 381 (8.4%) were females with the male to female ratio of 11. Most of the patients admitted with LBRF- like symptoms were within age groups 11–20 years accounted for 74.2%. Majority of LBRF cases were from urban dwellers 4305 (94.4%) (Table 1). However, deaths were not recorded.

**Table 1:** Distribution of sociodemographic characteristics of patients admitted with LBRF- like symptoms at Felegehiwot referral hospital from 2009- 2012

**Characteristic**	**Frequency**	**Percent (%)**
**Age group in years**		
1-10	447	9.8
11-20	3388	74.3
21-30	603	13.2
31-40	107	2.3
≥ 41	14	0.3
**Total**	**4559**	**100**
**Sex**		
Male	4178	91.6
Female	381	8.4
**Total**	**4559**	**100**
**Residence**		
Urban	4305	94.4
Rural	254	5.6
**Total**	**4559**	**100**

The overall prevalence of LBRF was 225 (4.9%). Highest 171 (5.1%) prevalence was found in age groups of 11–20. More male cases were recorded 207 (5%) than females 18 (4.7). Regards to the residence, more cases of urban dwellers 214 (5%) were recorded than rural residents 11 (4.3%). However, statistically significant association was not observed for: age, sex and residence of patients’ admitted with LBRF- like symptoms and prevalence of LBRF (P > 0.05) (Table 2).

**Table 2:** Effect of prevalence of louse-borne relapsing fever on sociodemographic characteristics of patients admitted with LBRF- like symptoms and seasonal variation at Felegehiwot referral hospital from 2009–2012

**Characteristic**	**Prevalence of louse-borne relapsing fever**			**P value**
	*LBRF positive	**LBRF negative**	**Total**	
	**No (%)**	**No (%)**	**No (%)**	
**Age group in years**				P < 0.861
1-10	22 (4.9)	425 (95.1)	447 (9.8)	
11-20	171 (5.1)	3217 (94.9)	3388 (74.3)	
21-30	28 (4.6)	575 (95.3)	603 (13.2)	
31-40	4 (3.7)	103 (96.2)	107 (2.3)	
≥ 41	0	14 (100)	14 (0.3)	
**Total**	**225 (4.9)**	**4334 (95.1)**	**4559 (100)**	
**Sex**				P < 0.843
Male	207 (5)	3971 (95)	4178 (91.6)	
Female	18 (4.7)	363 (95.3)	381 (8.4)	
**Total**	**225 (4.9)**	**4334 (95.1)**	**4559 (100)**	
**Residence**				P < 0.647
Urban	214 (5)	4091 (95)	4305 (94.4)	
Rural	11 (4.3)	243 (95.7)	254 (5.6)	
**Total**	**225 (4.9)**	**4334 (95.1)**	**4559 (100)**	
**Seasonal variation**				P < 0.001
Dry	27 (9.7)	251 (90.3)	278 (6.1)	
Wet	198 (4.6)	4083 (95.4)	4281 (93.9)	
**Total**	**225 (4.9)**	**4334 (95.1)**	**4559 (100)**	

*LBRF: - Louse-borne relapsing fever positive by microscopy for *Borellia* spp.

**Figure 1:**
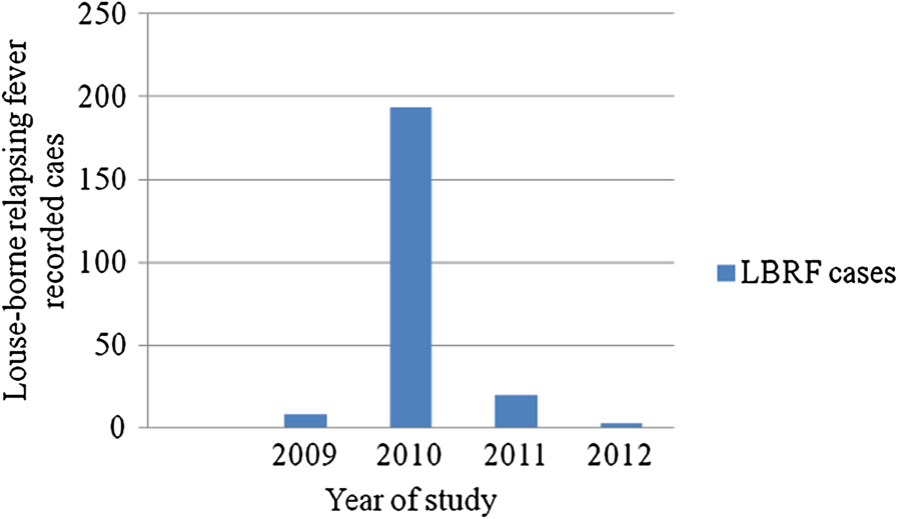
Trends in prevalence of louse-borne relapsing fever at Felegehiwot referral hospital Bahir Dar city for the last four years, 2009- 2012.

An attempt had been made to determine the association between seasonal variation and prevalence of LBRF. Statistically significant association was observed between prevalence of LBRF and seasons thus more patients positive for *Borrelia* species were recorded in dry 27 (9.7%) than wet 198 (4.6%) seasons ( P < 0.001) (Table 2). A trend in prevalence of LBRF for the last four years showed that the highest numbers of cases were recorded in 2010. Whereas the least cases were documented in 2012 (Figure 1).

## Discussion

In this study, the overall prevalence of LBRF was higher than the study done in Hossana hospital in Southern Ethiopia [2] and Jimma hospital in South western Ethiopia [12]. This difference might be due to the occurrence of LBRF epidemic in 2010 in Bahir Dar city [data from the registration book].

The highest prevalence 171 (5.1%) was observed in age groups of 11–20 (Table 2). This increment in prevalence might be explained as most of the high risk populations were street children and yekolotemaries categorized in this age group, having limited information towards LBRF prevention [15]. More male cases were recorded 207 (5%) than females 18 (4.7%) (Table 2). This study was in line with the study done in Jimma hospital [12] and this might be because of more internal migration of males from rural to urban as a daily labourer and hence more chance of contracting the disease.

Regards to the residence, more cases of urban dwellers 214 (5%) were recorded than rural residents 11 (4.3%). This is because, high risk populations were live in the Bahir Dar city [15] and this increase more number of cases recorded in urban than rural residents. However, statistically significant association was not observed for: age, sex and residence with prevalence of LBRF (P > 0.05) (Table 2).

Moreover, the association between seasonal variation and prevalence of LBRF showed that more patients positive for *Borrelia* species were recorded in dry than wet seasons. This study was in agreement with the study done in Jimma hospital [12] and this might be due to more migration of high risk populations to the city in the dry than wet seasons [15]. Furthermore, a trend in prevalence of LBRF for the last four years showed that the highest numbers of cases were recorded in 2010. This might be described as in 2010 there was LBRF epidemic occurrence in the city [15]. In contrast, the least numbers of cases were recorded in 2012 (Figure 1). This might be because of delivering of health education towards LBRF prevention in the city and this might decrease the numbers of recorded cases.

Since this study was done retrospectively from hospital recorded cases of LBRF and hence limited to address the real figure in the prevalence of LBRF in the study area. Therefore, further study at the community level should be done to determine the actual prevalence.

## Conclusion

This assessment revealed that louse-borne relapsing fever is one of the major public problems in the study area affecting young age groups of the population. Therefore, health education should be delivered towards LBRF prevention in the city.

## Competing interests

The authors declare that they have no competing interests.

## Authors’ contributions

MY carried out drafting the proposal, involved in data collection and analysis up to final submission. BA carried out proposal review, data analysis and critically reviewed the manuscript up to final submission. WM participated in proposal review, data analysis and final result review up to final submission. WA participated in proposal review and final result review up to final submission. All authors read and approved the final manuscript.

## Authors’ information

MY is a lecturer at College of Medicine and Health Sciences, Bahir Dar University in Medical Parasitology and head of Medical Parasitology. BA is an associate professor at College of Medicine and Health Sciences, Bahir Dar University in Medical Microbiology and department head of Microbiology, Immunology and Parasitology. WM is lecturer at College of Medicine and Health Sciences, Bahir Dar University in Medical Microbiology. WA is a Medical laboratory technologist at Felegehiwot referral hospital, Amhara National Regional State health Bureau.
